# Molecular Crosstalk of Vitamin D3 with cGAS–STING and BDNF Pathways in a Rat Model of Chronic Stress

**DOI:** 10.3390/ijms262110436

**Published:** 2025-10-27

**Authors:** May M. Alrashed, Hajera Tabassum, Dara Aldisi, Mahmoud M. A. Abulmeaty

**Affiliations:** 1 Chair of Medical and Molecular Genetics Research, Department of Clinical Laboratory Sciences, College of Applied Medical Sciences, King Saud University, P.O. Box 10219, Riyadh 11362, Saudi Arabia; hshareef@ksu.edu.sa; 2 Department of Community Health Sciences, College of Applied Medical Sciences, King Saud University, P.O. Box 10219, Riyadh 11362, Saudi Arabia; daldisi@ksu.edu.sa (D.A.); mabulmeaty@ksu.edu.sa (M.M.A.A.)

**Keywords:** vitamin D3, cGAS–STING pathway, BDNF, VDR, chronic unpredictable mild stress, neuroinflammation, hippocampus

## Abstract

Neuroinflammation via the cyclic GMP-AMP synthase (cGAS)–stimulator of interferon genes (STING) pathway and reduced hippocampal brain-derived neurotrophic factor (BDNF) expression are key mechanisms underlying stress-induced depression. Vitamin D3, acting through the vitamin D receptor (VDR), is known to possess immunomodulatory and neurotrophic properties, but its role under chronic stress remains unclear. This study investigated the effects of vitamin D3 on chronic unpredictable mild stress (CUMS)-induced neuroinflammation and neurotrophic deficits in male Wistar rats. Thirty-two rats were divided into four groups: control, CUMS only, CUMS + vitamin D3 (1000 IU/kg), and CUMS + vitamin D3 (10,000 IU/kg). Vitamin D3 was injected intramuscularly three times weekly for 28 days. Hippocampal mRNA expression of cGAS–STING pathway markers, BDNF, microglial activation marker Iba1, and pro-inflammatory cytokines was quantified by RT-qPCR, and relative expression was calculated using the 2^−ΔΔCt^ method. Serum vitamin D3 and corticosterone concentrations were measured by ELISA. CUMS significantly reduced serum vitamin D3, increased corticosterone, activated hippocampal cGAS–STING signaling, upregulated inflammatory mediators and Iba1, and suppressed VDR and BDNF mRNA expression (all *p* < 0.05). Vitamin D3 administration effectively restored serum vitamin D3, normalized corticosterone levels, attenuated cGAS–STING activation and inflammatory gene expression, reduced microglial activation, and enhanced hippocampal VDR and BDNF mRNA expression (all *p* < 0.05). These findings demonstrate that vitamin D3 alleviates CUMS-induced hippocampal inflammation and neurotrophic deficits through coordinated modulation of immune signaling and BDNF, highlighting its potential as a therapeutic approach for stress-related brain disorders.

## 1. Introduction

Stress is a major risk factor for the development of psychiatric disorders, particularly depression. Prolonged or unpredictable stress disrupts neural homeostasis, leading to emotional and cognitive impairments that mirror key features of Major Depressive Disorder (MDD), a condition affecting over 280 million people worldwide [1,2]. Chronic Unpredictable Mild Stress (CUMS) in rodents is a widely used preclinical paradigm that reliably models the effects of sustained stress, inducing behavioral, neuroinflammatory, and neurotrophic alterations relevant to human depression [3,4]. Among brain regions, the hippocampus is especially vulnerable, with stress exposure impairing its role in memory, learning, and emotional regulation [5,6].

A hallmark consequence of chronic stress is hippocampal neuroinflammation, which disrupts neuroplasticity and contributes to the progression of mood disorders [7]. Recent evidence highlights the cyclic GMP–AMP synthase (cGAS)–stimulator of interferon genes (STING) pathway as a pivotal driver of stress-induced neuroinflammatory responses [7,8,9]. Activation of cGAS–STING initiates downstream signaling through TANK-binding kinase 1 (TBK1) and interferon regulatory factor 3 (IRF3), culminating in the production of pro-inflammatory cytokines such as TNF-α, IL-6, IFN-γ, and NF-κB [8,9,10]. Persistent activation of this pathway has been implicated in hippocampal dysfunction under chronic stress conditions [10]. Concurrently, brain-derived neurotrophic factor (BDNF), a central regulator of neuronal survival, synaptic plasticity, and hippocampal neurogenesis, is consistently downregulated in chronic stress and CUMS paradigms [6]. This decline in BDNF weakens neurotrophic support and contributes to structural and functional impairments in the hippocampus. While hippocampal BDNF suppression is strongly associated with neuroinflammation and cGAS–STING pathway activation, additional mechanisms including glucocorticoid excess, oxidative stress, and epigenetic modifications also contribute to reduced neurotrophic signaling under chronic stress [11,12]. In parallel, chronic stress drives microglial cells toward a pro-inflammatory M1 phenotype, reflected by elevated expression of ionized calcium-binding adapter molecule 1 (Iba1) and increased secretion of cytokines such as TNF-α and IL-6, further amplifying neuronal injury and synaptic dysfunction [12,13,14].

Given these mechanisms, novel therapeutic strategies are needed to target both neuroinflammation and neurotrophic deficits. Conventional antidepressants remain limited by variable efficacy and adverse effects, encouraging exploration of nutritional and neuroactive compounds [15,16,17]. Among these, vitamin D3 has gained attention due to its pleiotropic actions [18]. Beyond its classical roles, vitamin D3 functions as a neuroactive steroid, with vitamin D receptors (VDRs) expressed in hippocampal and mood-regulating brain regions [19]. Clinical studies largely link vitamin D3 deficiency with higher depression risk, although the effects of supplementation in individuals with sufficient levels remain inconsistent, particularly in complex disorders such as major depressive disorder [18,20]. Preclinical evidence indicates that vitamin D3 can suppress inflammatory signaling and enhance BDNF expression. However, its effects on stress-induced hippocampal molecular pathways, including cGAS–STING activation, VDR modulation, and microglial activity, remain poorly understood [18,21,22]. The present study was therefore designed to evaluate the effects of vitamin D3 on hippocampal molecular alterations in CUMS-exposed rats. Specifically, we investigated whether vitamin D3 supplementation can suppress cGAS–STING signaling, restore BDNF expression, and modulate microglial activation. To our knowledge, this is the first study to provide direct molecular evidence for vitamin D3’s regulatory role in these pathways within a CUMS model, underscoring its translational potential as a therapeutic intervention for stress-related hippocampal dysfunction.

## 2. Result

### 2.1. Body Weight Changes, Serum Vitamin D_3_, and Corticosterone Levels in Different Groups

**Figure 1:**
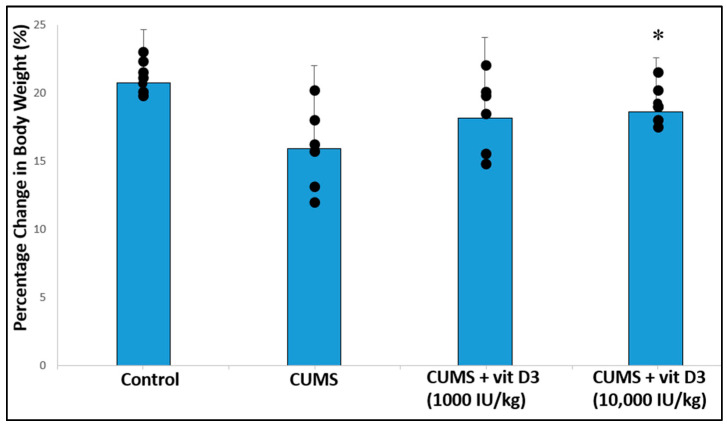
Percentage change in body weight relative to baseline in different groups. Values are presented as mean ± SD (*n* = 8 per group). Individual rat values are shown as dots. Statistical analysis was performed using one-way ANOVA followed by Holm–Sidak test. * *p* < 0.05 for vitamin D3 treated group versus CUMS (Bonferroni-corrected).

Vitamin D3 supplementation significantly affected absolute body weight in CUMS-exposed rats (*p* < 0.05). However, when body weight was analyzed as percentage change relative to baseline, the differences between groups were not statistically significant. As shown in Figure 1, CUMS reduced body weight gain compared to control rats (CUMS: 16.22 ± 16.20% vs. control: 21.12 ± 8.63%), although this difference did not reach statistical significance. Treatment with Vitamin D3 at 1000 IU/kg (18.51 ± 12.13%) did not significantly alter body weight relative to the CUMS group. In contrast, the high-dose Vitamin D3 group (10,000 IU/kg; 19.13 ± 1.25%) showed a significant increase in body weight compared to CUMS after Bonferroni correction (*p* < 0.05). Overall, these results indicate that high-dose Vitamin D3 may partially mitigate CUMS-induced body weight reduction, while low-dose supplementation has no significant effect.

**Figure 2:**
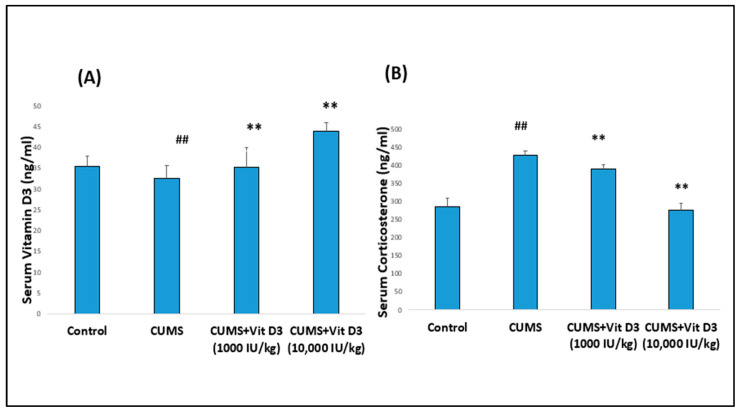
Serum (**A**) Vitamin D3 and (**B**) Corticosterone levels in control and CUMS-exposed rats with vitamin D3 supplementation. Values are presented as mean ± SD (*n* = 8 per group). Statistical analysis was performed using one-way ANOVA followed by Holm–Sidak post hoc tests. ^##^ indicates *p* < 0.001 for CUMS versus the control group, and ** indicates *p* < 0.001 for CUMS versus the vitamin D3 treated groups.

Similarly, serum Vitamin D3 levels were markedly reduced in CUMS-exposed rats compared with controls (32.52 ± 3.10 vs. 35.33 ± 2.50 ng/mL; *p* < 0.001) (Figure 2A). In the CUMS + vitamin D3 (1000 IU/kg) group, serum vitamin D3 levels were restored to near-control values (35.17 ± 4.80 ng/mL; *p* < 0.001 compared with CUMS). The CUMS + vitamin D3 (10,000 IU/kg) exhibited a more pronounced increase (43.86 ± 1.93 ng/mL), which was significantly higher than both the control and CUMS groups (*p* < 0.001). In contrast, CUMS exposure significantly elevated serum corticosterone levels relative to controls (426.31 ± 13.06 vs. 283.86 ± 23.88 ng/mL; *p* < 0.001) (Figure 2B). Treatment with Vitamin D3 at 1000 IU/kg partially attenuated this increase (390.08 ± 10.93 ng/mL; *p* < 0.001 compared with CUMS), while the higher dose (10,000 IU/kg) restored corticosterone levels to near-control values (276.30 ± 17.55 ng/mL; *p* < 0.001 compared with CUMS) (Table S1).

### 2.2. Vitamin D3 Attenuates CUMS-Induced Activation of the cGAS–STING Pathway in the Hippocampus

**Figure 3:**
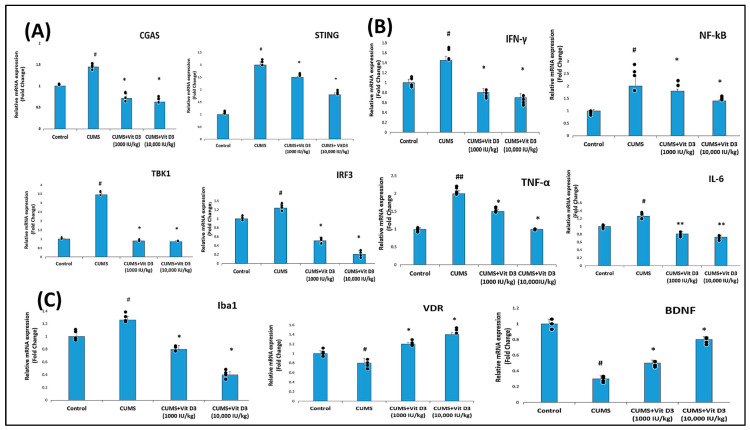
mRNA expression of cGAS–STING pathway genes, pro-inflammatory cytokines, microglial activation marker, and BDNF in the hippocampus of CUMS-exposed rats and the effect of vitamin D3 treatment. (**A**) Relative mRNA expression of cGAS, STING, TBK1, and IRF3 showing activation of the cGAS–STING pathway following CUMS exposure and attenuation by vitamin D3 in a dose-dependent manner. (**B**) Expression of IFN-γ, NF-κB, TNF-α, and IL-6 illustrating suppression of CUMS-induced pro-inflammatory cytokine upregulation by vitamin D3. (**C**) Expression of Iba1, VDR, and BDNF demonstrating reduction in microglial activation, restoration of VDR levels, and recovery of BDNF expression with vitamin D3 treatment. Data are presented as fold change relative to the control group (set at 1). Statistical analysis was performed using one-way ANOVA followed by Holm–Sidak post hoc tests. ^#^ indicates *p* < 0.05 and ^##^ *p* < 0.001 for CUMS versus control; * indicates *p* < 0.05 and ** *p* < 0.001 for vitamin D3-treated groups versus CUMS.

CUMS significantly upregulated key genes in the cGAS–STING pathway, with CGAS at 1.45 ± 0.086-fold (*p* < 0.05), STING at 3.00 ± 0.12-fold (*p* < 0.05), TBK1 at 3.46 ± 0.09-fold (*p* < 0.05), and IRF3 at 1.24 ± 0.09-fold (*p* < 0.05) relative to controls (set at 1), indicating activation of this innate immune signaling pathway in response to chronic stress (Figure 3A, Table S2). Vitamin D3 treatment attenuated this activation in a dose-dependent manner. Administration of Vitamin D3 (1000 IU/kg) partially reduced expression of CGAS (0.71 ± 0.12-fold), STING (2.5 ± 0.04-fold), TBK1 (0.89 ± 0.06-fold), and IRF3 (0.51 ± 0.08-fold; *p* < 0.05 vs. CUMS), while the higher dose of 10,000 IU/kg further suppressed expression, particularly CGAS (0.63 ± 0.08-fold) and IRF3 (0.21 ± 0.08-fold), with partial reductions in STING (1.80 ± 0.08-fold) and TBK1 (0.85 ± 0.008-fold; *p* < 0.05 vs. CUMS), effectively restoring these markers toward baseline control levels. These results demonstrate that Vitamin D3 suppresses CUMS-induced activation of the cGAS–STING pathway, with stronger inhibitory effects at higher doses, especially on CGAS and IRF3.

### 2.3. Vitamin D3 Suppresses CUMS-Induced Upregulation of Pro-Inflammatory Cytokines in the Hippocampus

CUMS significantly increased the expression of pro-inflammatory cytokines and NF-κB in the hippocampus (Figure 3B). Relative to controls (set at 1-fold), CUMS elevated IFN-γ to 1.45 ± 0.085-fold (*p* < 0.05), NF-κB to 2.00 ± 0.4-fold (*p* < 0.05, TNF-α to 2.20 ± 0.4-fold (*p* < 0.001), and IL-6 to 1.26 ± 0.08-fold (*p* < 0.05). Vitamin D3 treatment attenuated these increases in a dose-dependent manner. Administration of Vitamin D3 (1000 IU/kg) reduced IFN-γ to 0.80 ± 0.08-fold (*p* < 0.05 vs. CUMS), NF-κB to 1.80 ± 0.08-fold (*p* < 0.05 vs. CUMS), TNF-α to 1.50 ± 0.08-fold (*p* < 0.001 vs. CUMS), and IL-6 to 0.81 ± 0.085-fold (*p* < 0.001 vs. CUMS). The higher dose of 10,000 IU/kg further suppressed expression, with IFN-γ at 0.70 ± 0.083-fold (*p* < 0.05 vs. CUMS), NF-κB at 1.40 ± 0.04-fold (*p* < 0.05 vs. CUMS), TNF-α at 1.00 ± 0.008-fold (*p* < 0.001 vs. CUMS), and IL-6 at 0.73 ± 0.083-fold (*p* < 0.001 vs. CUMS), demonstrating a dose-dependent mitigation of CUMS-induced neuroinflammation by Vitamin D3.

### 2.4. Vitamin D3 Reduces Microglial Activation Marked by Iba1 Expression in CUMS-Exposed Rats

Figure 3C shows that exposure to CUMS triggered pronounced microglial activation in the hippocampus, as indicated by a significant upregulation of Iba1 expression. Compared with controls (set at 1-fold), CUMS elevated Iba1 to 1.26 ± 0.06-fold (*p* < 0.05). Vitamin D3 treatment attenuated this activation in a dose-dependent manner, with 1000 IU/kg reducing Iba1 to 0.80 ± 0.06-fold (*p* < 0.05 vs. CUMS) and 10,000 IU/kg further suppressing it to 0.40 ± 0.12-fold (*p* < 0.05 vs. CUMS), demonstrating the role of vitamin D3 to mitigate stress-induced microglial activation.

### 2.5. Vitamin D Receptor (VDR) Expression Is Modulated in Response to CUMS and Vitamin D3

Hippocampal VDR expression was significantly reduced in the CUMS group compared with controls (mean fold change: 0.90 ± 0.20 vs. 1.0 ± 0.0, *p* < 0.05), indicating stress-induced suppression of the vitamin D receptor (Figure 3C). Vitamin D3 treatment markedly enhanced VDR expression in a dose-dependent manner. The CUMS group treated with 1000 IU/kg showed a significant increase (1.20 ± 0.04), while the higher dose 10,000 IU/kg further restored expression (1.30 ± 0.04, *p* < 0.001 vs. CUMS), approaching or exceeding control levels.

### 2.6. Vitamin D3 Restores BDNF Expression in the Hippocampus

CUMS profoundly impaired hippocampal neurotrophic support, as reflected by a significant downregulation of BDNF expression. Compared with controls (set at 1-fold), CUMS reduced BDNF to 0.30 ± 0.04-fold (*p* < 0.05) (Figure 3C). Vitamin D3 treatment attenuated this decrease in a dose-dependent manner, restoring BDNF to 0.50 ± 0.04-fold (*p* < 0.05 vs. CUMS) at 1000 IU/kg and to 0.80 ± 0.04-fold (*p* < 0.05 vs. CUMS) at 10,000 IU/kg, demonstrating its ability to counteract stress-induced neurotrophic deficits.

## 3. Discussion

This study identifies four key hallmarks of CUMS-induced hippocampal pathology: activation of the cGAS–STING pathway, enhanced pro-inflammatory and microglial responses, suppression of BDNF signaling, and their partial reversal by vitamin D3 supplementation. CUMS significantly increased hippocampal expression of cGAS, STING, TBK1, IRF3, and Iba1 while reducing VDR and BDNF levels. Vitamin D3 counteracted these changes in a dose-dependent manner, with the higher dose producing stronger suppression of neuroinflammatory markers and more robust restoration of BDNF. These findings position vitamin D3 as a potential modulator of stress-induced neuroinflammation and impaired neuroplasticity.

Vitamin D is increasingly recognized for its role in brain health beyond calcium and bone regulation. Hippocampal and prefrontal cortical neurons and glia express VDR, which influences neurotrophic support, synaptic function, and immune responses. In the present study, Vitamin D3 supplementation restored serum vitamin D3 levels (Figure 2; Table S1) and hippocampal VDR mRNA expression (Figure 3C), suggesting improved receptor-mediated signaling and stabilization of HPA axis activity, as reflected by normalized corticosterone levels. These findings align with previous reports; for instance, Al-Ramadhan et al., 2023 showed that Vitamin D3 supplementation counteracted CUMS-induced molecular alterations, suggesting potential relevance to the amelioration of depression-related mechanisms [17]. Additionally, these experimental results complement clinical observations linking vitamin D deficiency with depressive symptoms [18,23] and recent studies report a high prevalence of vitamin D3 deficiency in depressed individuals, highlighting its potential as an adjunctive therapeutic strategy [24]. CUMS exposure markedly activated the cGAS–STING pathway in the hippocampus, as indicated by elevated CGAS, STING, TBK1, and IRF3 expression (Figure 3A). The cGAS–STING pathway functions as a central innate immune mechanism, detecting cytosolic DNA and initiating inflammatory signaling via TBK1–IRF3 and NF-κB activation. Upon DNA recognition, cGAS generates cyclic GMP–AMP (cGAMP), which activates STING, recruits TBK1, phosphorylates IRF3, and induces type I interferons alongside pro-inflammatory cytokines [19]. This hyperactivation in our CUMS model is consistent with previous studies showing persistent cGAS–STING activation drives neuroinflammation and hippocampal dysfunction in stress-related and neurodegenerative conditions [8,10,19,20,21]. Notably, vitamin D3 is reported to suppress STING-induced interferon production and downstream inflammatory mediators in various models [22,25]. Collectively, these findings support the role of vitamin D3 as a modulator of cGAS–STING signaling, likely through indirect mechanisms involving VDR-mediated anti-inflammatory and neurotrophic actions, consistent with the attenuation of neuroinflammation.

Vitamin D3 treatment reduced IFN-γ, TNF-α, IL-6, and NF-κB mRNA expression in a dose-dependent manner (as shown in Figure 3B), indicating dual anti-inflammatory effects: suppression of upstream cGAS–STING signaling and attenuation of downstream cytokine release. These findings align with earlier reports identifying NF-κB driven neuroinflammation as a hallmark of chronic stress and a key contributor to depressive pathology. Consistent with our results, Li et al., 2019 reported elevated TNF-α, IL-6, and IL-1β in the brains of CUMS-exposed rats, while White et al., 2024 demonstrated NF-κB driven neuroinflammation in both rodent and human models of chronic stress [26,27]. Similarly, Sălcudean et al., 2025 emphasized aberrant NF-κB activation and cytokine overproduction as central drivers of depression pathophysiology [7]. Importantly, NF-κB appeared particularly sensitive to vitamin D3 treatment in our study, echoing evidence from Wang et al., 2021 showing vitamin D3-mediated NF-κB inhibition [28]. Together, these results strengthen the view that vitamin D3 mitigates stress-induced hippocampal inflammation by targeting both upstream cGAS–STING and downstream NF-κB–cytokine pathways [22,25].

Similarly, CUMS induced a moderate increase in Iba1 expression (Figure 3C), reflecting microglial activation in response to chronic stress. Vitamin D3 partially suppressed this increase, suggesting a trend toward reduced neuroimmune activation. Microglia, as the resident immune cells of the central nervous system, play a pivotal role in orchestrating hippocampal inflammation by releasing pro-inflammatory cytokines such as TNF-α and IL-6. The attenuation of Iba1 expression by vitamin D3 aligns with its inhibitory effects on upstream cGAS–STING signaling and downstream NF-κB mediated cytokine production, highlighting a coordinated modulation of both microglial activity and inflammatory signaling under chronic stress conditions [29]. Supporting this, Li et al., 2019 demonstrated that CUMS-induced hippocampal microglial activation was accompanied by increased pro-inflammatory cytokines [26], while Han et al., 2023 further highlighted microglial dysfunction as a critical mediator of stress-related depression [30]. Importantly, inhibition of cGAS signaling has been shown to reduce both microglial activation and cytokine release, linking vitamin D3’s regulation of the cGAS–STING pathway to its capacity to modulate microglial-driven neuroinflammation [26,27,31]. Vitamin D3 supplementation reduced Iba1 expression, indicating a potential decrease in microglial activation.

Interestingly, vitamin D3 was found to reverse CUMS-induced hippocampal BDNF suppression in a dose-dependent manner (Figure 3C), highlighting its role in sustaining neuroplasticity under stress. These findings are consistent with previous studies demonstrating vitamin D3-mediated enhancement of hippocampal BDNF and neurogenesis [22,32,33,34,35]. Wu et al., 2025 reported that CUMS disrupts the PGC-1α/FNDC5/BDNF axis, while Han et al., 2018 linked reductions in BDNF and synaptic plasticity markers to immune activation and depressive-like behaviors [22,32]. Similarly, Xu and Liang (2021) showed that Vitamin D3/VDR signaling mitigates post-stroke depression by upregulating hippocampal BDNF [29], and Numakawa and Kajihara (2025) emphasized that impaired BDNF–TrkB signaling is a hallmark of depression, schizophrenia, and neurodegeneration [6]. Restoration of this pathway is therefore critical for neuronal survival, differentiation, and synaptic plasticity. In line with this framework, our results suggest that vitamin D3 enhances hippocampal resilience by reinstating BDNF signaling, paralleling the effects of nutraceuticals such as resveratrol and curcumin, which also alleviate stress-induced pathology via BDNF upregulation [34,35]. While hippocampal BDNF suppression in this study appears primarily linked to neuroinflammation and cGAS–STING activation, it is important to acknowledge that stress-induced BDNF loss is multifactorial. In addition to these mechanisms, elevated glucocorticoids, epigenetic modifications, and oxidative stress, may also contribute to reduced neurotrophic support under chronic stress [11,12].

**Figure 4:**
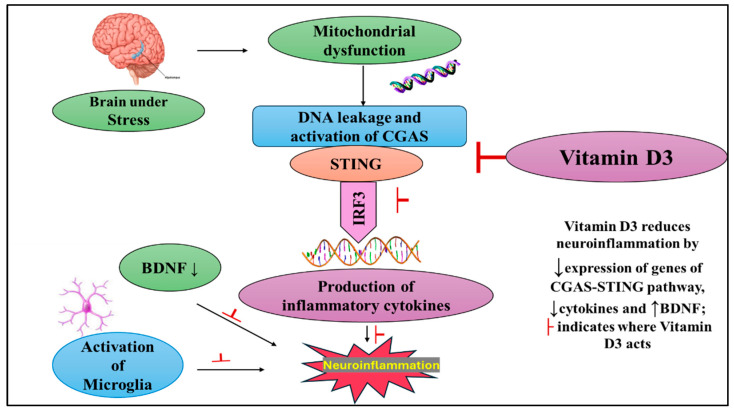
Schematic representation of the cGAS–STING pathway in CUMS-induced neuroinflammation and its modulation by Vitamin D3. Chronic stress triggers cGAS–STING signaling, elevating pro-inflammatory cytokines and activating microglia, resulting in reduced BDNF expression and neuronal impairment. Vitamin D3 counteracts these effects by suppressing cGAS–STING activation, limiting inflammation, and restoring BDNF levels. (↑) indicate an increase or activation, whereas (↓) indicate a decrease or suppression and T-bar symbols (├) represent inhibition.

A proposed mechanism underlying these findings is illustrated in Figure 4. Mechanistically, chronic stress induces sustained oxidative and mitochondrial dysfunction in hippocampal neurons, leading to the release of mitochondrial DNA into the cytosol. This DNA activates cGAS, triggering cGAMP production and STING signaling, which subsequently engages TBK1 and IRF3 to drive NF-κB activation and the release of pro-inflammatory cytokines [10]. The resulting neuroinflammatory milieu suppresses BDNF expression, thereby compromising neuronal survival and plasticity [32]. Vitamin D3 counters these pathological processes by enhancing hippocampal VDR expression, strengthening vitamin D signaling. Activated VDR complexes inhibit cGAS–STING and NF-κB activity, reduce cytokine release, and restore BDNF signaling [32]. Through this integrated regulation of immune and neurotrophic pathways, vitamin D3 presumably alleviates neuroinflammation, preserves hippocampal function, and enhances resilience to chronic stress.

A limitation of the present study is the reliance on mRNA expression without protein-level confirmation for key targets such as BDNF, VDR, and cGAS–STING pathway components. While transcriptional changes provide mechanistic insights, mRNA levels do not always reflect protein abundance or activity. This limitation was partly due to the small amount of hippocampal tissue and restricted resources. Additionally, although our findings suggest that hippocampal BDNF suppression is primarily linked to neuroinflammation and cGAS–STING activation, other mechanisms such as elevated glucocorticoids, epigenetic modifications, and oxidative stress may also contribute to reduced neurotrophic support under chronic stress. These pathways were not directly examined in the present study but warrant further investigation. Another limitation is that the work was conducted in a rodent model, and extrapolation to human depression should be made with caution. Future studies incorporating protein-level assays, behavioral validation, and sex-based comparisons will further clarify the mechanistic and translational relevance of vitamin D3 as a modulatory strategy in stress-related brain disorders.

## 4. Materials and Methods

### 4.1. Animals, Study Design, and Ethics

In this study, thirty-two adults male Wistar rats (8–10 weeks old, weighing 200–250 g) were used. Animals were housed in the Biomedical Animal Research Unit, College of Applied Medical Sciences, King Saud University, under standard laboratory conditions. The animal study protocol was approved by the Animal Research Ethics Committee of the King Saud University (Ref. No. KSU-SE-22-24) dated 24 March 2022.

#### 4.1.1. Animal Groups

The rats were randomly assigned into groups of eight, with the sample size determined by power analysis using G*Power (v3.1.9.4), assuming an effect size of 0.8, α = 0.05, and 95% statistical power. The rats were housed in cages, maintained under standard laboratory conditions, including a 12 h light/dark cycle at a room temperature of 25 ± 2 °C, with free access to standard rodent chow and water.

Group I (Control group): This group included rats that received intramuscular saline injections and were not subjected to any stress.

Group II (CUMS only): This group consists of rats exposed to a CUMS protocol for 28 consecutive days without receiving any vitamin D3 treatment.

Group III (CUMS + Vit D3 1000 IU/kg): This group consists of rats that underwent the same 28-day CUMS protocol as Group II and received vitamin D3 at a dose of 1000 IU/kg, intramuscularly (IM).

Group IV (CUMS + Vit D3 10,000 IU/kg) also underwent the CUMS protocol and received vitamin D3 at a higher dose of 10,000 IU/kg, IM.

#### 4.1.2. Chronic Unpredictable Mild Stress (CUMS) Induction: Protocol

Rats in Groups II, III, and IV were subjected to a CUMS protocol over 28 consecutive days. In this study, a CUMS model was established with minor modifications to previously described protocols [36]. Rats were exposed to a variety of mild, randomly scheduled stressors, including cage tilting, cage shaking, wet bedding, continuous light exposure, and food or water deprivation, to induce depression-like behaviors [33]. The sequence of stressors applied across the 28-day protocol is outlined in Table 1. The model was validated using behavioral tests (Sucrose Preference, Forced Swimming, Tail Suspension, Open Field, and Elevated Plus Maze), as previously reported [17], along with serum corticosterone measurements.

#### 4.1.3. Vitamin D3 Administration

Vitamin D3 was obtained from Memphis Company for Pharmaceutical and Chemical Industries (Cairo, Egypt; 2 mL ampule, 100,000 IU/mL). According to the manufacturer’s data, the medication includes Polysorbate 80 and propylene glycol to ensure optimal delivery and stability of vitamin D3. The required dose for each treatment group was prepared by dilution with 0.9% physiological saline (NaCl) [37,38]. The solution was administered intramuscularly to the rats in Groups III and IV three times per week throughout the 28-day CUMS protocol. Group III received a low dose of vitamin D3 (1000 IU/kg), while Group IV received a high dose (10,000 IU/kg). The selected doses of Vit D3 were according to the previously published work [39]. We preferred the IM route for efficient delivery of vitamin D3 [40]. The calculated dose for each rat was given every other day (Sundays, Tuesdays, and Thursdays). Sunday’s and Tuesday’s injections include a dose of 2 days, while those of Thursdays include a dose sufficient for 3 days to cover the weekend days. The control group was also injected with an equivalent volume of sterile saline.

### 4.2. Body Weight Measurement

The body weight of each rat was recorded at baseline and subsequently monitored at regular intervals using a calibrated electronic scale. Monitoring changes in body weight served as an indirect measure of overall health and physiological stress, providing additional insight into the effects of depressive-like behavior and treatment interventions during the experimental period.

### 4.3. Sample Collection

At the end of the experimental period, rats were euthanized under anesthesia (isoflurane anesthesia (Abbott, Wiesbaden, Germany)). During induction, isoflurane 2–3.5% was inhaled in 100% oxygen, while during anesthesia maintenance, the isoflurane dose was 1.5%. Blood was collected via cardiac puncture, centrifuged at 3500 rpm for 10 min, and the resulting serum was stored at −80 °C until analysis. Brains were rapidly dissected, and hippocampal tissues were isolated and preserved at −80 °C for subsequent molecular assessments.

### 4.4. Serum Analysis

Serum vitamin D3 and corticosterone were quantified using commercially available ELISA kits, following the manufacturers’ protocols.

#### 4.4.1. Serum Vitamin D3

Serum vitamin D3 were measured using a Rat 25 Dihydroxy Vitamin D ELISA Kit (My BioSource, San Diego, CA, USA; catalog number MBS2601819) according to the manufacturer’s instructions. Briefly, serum samples and standards were added to antibody-coated wells and incubated with vitamin D–HRP conjugate. After washing, substrate solution was added, and the resulting color, inversely proportional to vitamin D concentration, was measured at 450 nm and expressed in ng/mL.

#### 4.4.2. Serum Corticosterone

Corticosterone levels in serum were determined using Rat Corticosterone ELISA Kit (My BioSource, San Diego, CA, USA; catalog number MBS761865) following the manufacturer’s instructions. Serum samples and standards were incubated in antibody-coated wells with a corticosterone–HRP conjugate. After washing, a substrate solution was added, and the resulting color, inversely related to corticosterone concentration, was measured at 450 nm. Concentrations were calculated from a standard curve and reported in ng/mL.

### 4.5. Gene Expression Analysis by Quantitative Real-Time PCR (qRT-PCR)

#### 4.5.1. Tissue Source

The hippocampal tissues stored at −80 °C were used for gene expression analysis.

#### 4.5.2. RNA Extraction

Total RNA was extracted from hippocampal tissue samples using the PureLink™ RNA Mini Kit (Invitrogen, Thermo Fisher Scientific, Pleasanton, CA, USA) according to the manufacturer’s instructions. Tissue homogenization was performed on ice to preserve RNA integrity. Briefly, hippocampal tissue samples were homogenized in lysis buffer, followed by binding of RNA to a silica-based spin column. The column was washed to remove contaminants, and RNA was eluted in RNase-free water. RNA concentration and purity were assessed spectrophotometrically, using a Nano Drop spectrophotometer 2000 (Thermoscientific, Pleasanton, CA, USA), and only samples with an A260/A280 ratio between 1.8 and 2.0 were used for downstream applications. Extracted RNA was stored at –80 °C until further use.

#### 4.5.3. Reverse Transcription

High-Capacity cDNA Reverse Transcription Kit (Applied Biosystems, Pleasanton, CA, USA) was used for cDNA synthesis. Total RNA (1 µg) was reverse transcribed in a 20 µL reaction volume under the manufacturer’s recommended thermal cycling conditions (25 °C for 10 min, 37 °C for 120 min, and 85 °C for 5 min) using a Veriti Thermal Cycler (Applied Biosystems, Thermo Fisher Scientific, Pleasanton, CA, USA). The resulting cDNA was stored at −20 °C until analysis.

#### 4.5.4. Quantitative Real-Time PCR

Quantitative real-time PCR (qPCR) was carried out using Power SYBR™ Green PCR Master Mix (Applied Biosytems, Thermoscientific, Pleasanton, CA, USA) on a Rotor-Gene Q Real-Time PCR System (Qiagen, Hilden, Germany). Each 20 µL PCR reaction contained 1 µL of cDNA, 10 µL of SYBR Green master mix, 1 µL each of forward and reverse primers (Macrogen, Seoul, Republic of Korea), and 7 µL of nuclease-free water. All reactions were performed in duplicate. The thermal cycling conditions included initial denaturation at 95 °C for 10 s, followed by 40 cycles of 95 °C for 10 s and 60 °C for 30 s. Melting curve analysis was conducted at the end of each run to confirm the specificity of amplification.

Target genes assessed by qPCR: For the cGAS–STING pathway, the genes analyzed included *cGAS*, *STING*, *TBK1*, and *IRF3*, which are key regulators of innate immune activation. To evaluate neuroinflammation, expression levels of major pro-inflammatory cytokines-*TNF-α*, *IL-6*, *IFN-γ*, and *NF-κB* were measured. The neurotrophic factor *BDNF* was included in assessing synaptic support and neuronal plasticity. Microglial activation was examined by quantifying the expression of *Iba1*, a marker of activated microglia. In addition, the expression of the vitamin D receptor (*VDR*) gene was measured to evaluate the involvement of vitamin D signaling in modulating stress-induced molecular changes. Primers sequences used for gene expression by qPCR are listed in Table 2. *GAPDH* served as the housekeeping gene for normalization. Relative gene expression was calculated using 2^−ΔΔCt^ method.

### 4.6. Statistical Analysis

All data were analyzed using Sigmaplot software version 12. One-way ANOVA followed by Holm–Sidak test used for group comparisons. A *p*-value < 0.05 was considered statistically significant.

## 5. Conclusions

In conclusion, these findings demonstrate that vitamin D3 mitigates CUMS-induced hippocampal neuroinflammation and enhances neurotrophic signaling. While the results suggest potential therapeutic relevance, they do not establish definitive clinical efficacy. Nonetheless, the study advances understanding of the interactions between vitamin D3, cGAS–STING signaling, microglial activation, and BDNF in stress-induced hippocampal dysfunction, providing a foundation for future preclinical and clinical investigation.

## Figures and Tables

**Table 1:** Various Mild Stressors Applied During the 28-Day CUMS Protocol.

	Week 1	Week 2	Week 3	Week 4
**Day 1**	Cage tilting at 45° for 24 h	Food deprivation for 24 h	Shaking the cage at 150 rpm for 1 h	Wet bedding for 24 h
**Day 2**	Shaking the cage at 150 rpm for 1 h	Wet bedding for 24 h	Cage tilting at 45° for 24 h	Food deprivation for 24 h
**Day 3**	Wet bedding for 24 h	Shaking the cage at 150 rpm for 1 h	Wet bedding for 24 h	Cage tilting at 45° for 24 h
**Day 4**	Water deprivation for 24 h	Cage tilting at 45° for 24 h	Water deprivation for 24 h	Shaking the cage at 150 rpm for 1 h
**Day 5**	Continuous light exposure for 24 h	Continuous light exposure for 24 h	Continuous light exposure for 24 h	Continuous light exposure for 24 h
**Day 6**	Continuous light exposure for 24 h	Continuous light exposure for 24 h	Continuous light exposure for 24 h	Continuous light exposure for 24 h
**Day 7**	Food deprivation for 24 h	Water deprivation for 24 h	Food deprivation for 24 h	Water deprivation for 24 h

**Table 2:** Primers sequences used for gene expression studies by qPCR.

Gene	Primer Sequence (5′–3′)
*CGAS*	F-AAGATCCGCGTAGAAGGACGA
	R-CTCCACGGTGACATCTGTATCT
*STING*	F-TAGCACTTCACATAGCCTCGC
	R-GAATGTGGGACTCATCTGGAG
*TBK1*	F-GCACATCAGGAAGGCACTCA
	R-CTTGTAGAGGAACTGACCCTAG
*IRF3*	F-TTCAGGATCCCATGGAAGCATG
	R-TTATTGGAGCAGCTGAGCTGG
*BDNF*	F-AGGGAAATCTCCTGAGCCGA
	R-TAATCCAATTTGCACGCCGC
*IFN-γ*	F-ATTCATGAGCATCGCCAAGTTC
	R-ATACTGCCTGCCTGAAGCTCTTGT
*TNF-α*	F-AGATGTGGAACTGGCAGAGG
	R-CCCATTTGGGAACTTCTCCT
*IL-6*	F-AGTTGCCTTCTTGGGACTGA
	R-ACAGTGCATCATCGCTGTTC
*NF-κB*	F-ACGATCTGTTTCCCCTCATCT
	R-TGCTTCTCTCCCCAGGAATA
*Iba 1*	F-CCTGTATGGCTTTCCCATCAC
	R-ATTAGAAGGTCCTCGGTCCCA
*VDR*	F-GCCCCTCATAAAGTTCCAGGTG
	R-GGATAGGCGGTCCTGAATGG
*GADPH*	F-TGCACCACCAACTGCTTAGC
	R-GGATGCAGGGATGATGATGTTCT

## Data Availability

The original contributions presented in this study are included in the article. Further inquiries can be directed to the corresponding author(s).

## Supplementary Materials

The supporting information can be downloaded at: https://www.mdpi.com/article/10.3390/ijms262110436/s1.

## Abbreviations

The following abbreviations are used in this manuscript:

CUMS	Chronic Unpredictable Mild Stress
cGAS	Cyclic GMP–AMP Synthase
STING	Stimulator of Interferon Genes
TBK1	TANK-binding kinase 1
IRF3	Interferon Regulatory Factor 3
BDNF	Brain-derived neurotrophic factor
TNF-α	Tumor Necrosis Factor-Alpha
IL-6	Interleukin-6
IFN-γ	Interferon-Gamma
NF-κB	Nuclear factor-kappa B
