# Supplementary material for: Molecular Crosstalk of Vitamin D3 with cGAS–STING and BDNF Pathways in a Rat Model of Chronic Stress
Source: Int J Mol Sci. 2025 Oct 27;26(21):10436. doi: 10.3390/ijms262110436 (PMC12608955; doi:10.3390/ijms262110436)
Supplement: Supplementary file 1 [file ijms-26-10436-s001.zip › ijms-3908111-supplementary.pdf]

Table S1. Serum Vitamin D3 and corticosterone levels in control and CUMS-exposed rats with vitamin D3 supplementation.

Parameter	Control (Mean ± SD)	CUMS (Mean ± SD)	CUMS + Vit D3 (1,000 IU/kg) (Mean ± SD)	CUMS + Vit D3 (10,000 IU/kg) (Mean ± SD)
**Vitamin D3 (ng/ml)**	35.33 ± 2.50	32.52 ± 3.10##	35.17 ± 4.80**	43.86 ± 1.93**
**Corticosterone (ng/ml)**	283.86 ±23.88	426.31 ± 13.06##	390.08 ± 10.93**	276.30± 17.55**

Values are presented as mean ± SD (n = 8 per group). Statistical analysis was performed using one-way ANOVA followed by Holm–Sidak post hoc tests. ## indicates p<0.001 for CUMS versus the control group, and **indicates p<0.001 for CUMS versus the vitamin D3 treated groups.

Table S2. mRNA expression data of cGAS–STING pathway genes, pro-inflammatory cytokines, microglial activation marker, and BDNF in the hippocampus of CUMS-exposed rats and the effect of vitamin D3 treatment

Gene	Control	CUMS	CUMS + Vit D3 (1,000 IU/kg)	CUMS + Vit D3 (10,000 IU/kg)
CGAS	1.00 ± 0.03	1.45 ± 0.09#	0.71 ± 0.12*	0.63 ± 0.08*
STING	1.00 ± 0.05	3.00 ± 0.12#	2.50 ± 0.04*	1.80 ± 0.08*
TBK1	1.00 ± 0.03	3.46 ± 0.09#	0.89 ± 0.06*	0.85 ± 0.05*
IRF3	1.00 ± 0.05	1.24 ± 0.09#	0.51 ± 0.08*	0.21 ± 0.08*
INF-γ	1.00± 0.07	1.45 ± 0.08#	0.80 ± 0.08*	0.70 ± 0.083*
NF-$\kappa\beta$	1.00 ± 0.05	2.00 ± 0.40#	1.80 ± 0.08*	1.40 ± 0.04*
TNF-α	1.00 ± 0.05	2.00 ± 0.09##	1.50± 0.06*	1.00 ± 0.008*
IL-6	1.00 ± 0.04	1.26 ± 0.08#	0.81 ± 0.06**	0.73 ± 0.05**
Iba1	1.00 ± 0.07	1.26 ± 0.06#	0.80 ± 0.06*	0.40 ± 0.05*
BDNF	1.00 ± 0.06	0.30 ± 0.04#	0.50 ± 0.04*	0.80 ± 0.04*
VDR	1.00 ± 0.06	0.80 ± 0.10#	1.20 ± 0.04*	1.40 ± 0.04*

Data are presented as fold change relative to the control group (set at 1). Values are presented as mean ± SD (n = 4 per group). Statistical analysis was performed using one-way ANOVA followed by Holm–Sidak post hoc tests. # indicates $p < 0.05$ and ## p < 0.001 for CUMS versus control; * indicates $p < 0.05$ and ** $p < 0.001$ for vitamin D3-treated groups versus CUMS.
